# Vaporized Hydrogen Peroxide Decontamination of N95 Respirators in a Dedicated Animal Research Facility for Reuse During a Novel Coronavirus Pandemic

**DOI:** 10.1177/1535676020936381

**Published:** 2020-09-01

**Authors:** F. Claire Hankenson, Mark Mauntel, Jamie Willard, Leslie Pittsley, William Degg, Niko Burnell, Alan Vierling, Stanley Griffis

**Affiliations:** ^1^ Campus Animal Resources, Michigan State University, East Lansing, MI, USA; ^2^ Department of Pathobiology and Diagnostic Investigation, College of Veterinary Medicine, Michigan State University, East Lansing, MI, USA; ^3^ Environmental Health and Safety, Office of Regulatory Affairs, Michigan State University, East Lansing, MI, USA; ^4^ Sparrow Health System, East Lansing, MI, USA; ^5^ Department of Supply Chain Management, Eli Broad College of Business, Michigan State University, East Lansing, MI, USA

**Keywords:** vaporized hydrogen peroxide, decontamination, animal facility, respirator, N95, personal protective equipment, COVID-19

## Abstract

**Introduction:**

During the COVID-19 pandemic, health care systems and safety providers have faced an unprecedented challenge of limited access to personal protective equipment (PPE) to conduct patient and public care. In federal emergencies, reuse of PPE after disinfection can occur by processes, like vaporized hydrogen peroxide (VHP), recommended by the Centers for Disease and Control and Prevention. We identified a vacant animal holding facility at our institution to repurpose into a regional VHP decontamination center.

**Methods:**

The facility is a multiroom, 20 000 ft^2^ building with control of HVAC to adjust to VHP conditional requirements. H_2_O_2_ was delivered to rooms using robotic HaloFoggers, dispersing H_2_O_2_ vapor and increasingly concentrated microdroplets as a fog for a timed period based on cubic footage of rooms.

**Results:**

Fogging cycles eliminated 6-log *Geobacillus stearothermophilus* up to 7 days postcycle. Functional efficacy of treated N95s was confirmed by fit tests of institutional personnel. Signage, process flow mapping, and training materials facilitated ease of workflow and adherence to safety expectations within the building.

**Discussion and Conclusion:**

Our study determined that a variety of N95 respirator types and sizes were able to be cleared of potential bacterial and viral agents using VHP in a controlled fog/dwell/exhaust cycle. This repurposed animal facility has the capacity to decontaminate up to 6700 respirators daily, which will address the predicted surge of COVID-19 cases in the state, and ultimately allow each respirator to be reused multiple times. There is no other public site in the region with our capacity to offset the continued supply chain issues for PPE needs.

## Introduction

An urgent need exists for access to personal protective equipment (PPE), specifically N95 (also referred to as FFR, filtering face piece respirators) respirators, for health care providers, first-line responders, and police, fire, and safety officials in response to the COVID-19 pandemic. News media stories continue to highlight the extreme difficulty in procurement of PPE, the seizure of PPE shipments prior to distribution, and the practice of extending use of single N95s for multiple shifts, if not days, by health workers. To contribute to the decontamination effort for PPE needed in health sites in proximity to our institution, we repurposed a vacant animal research housing facility to establish a center for application of vaporized hydrogen peroxide (VHP) to disinfect critical medical materials for their return into service.

The project was designed using an interdisciplinary approach between veterinary and medical experts, hospital partners (Health Systems A & B), and environmental safety, microbiology, and supply chain experts. Numerous studies conducted with VHP have evaluated its use in veterinary and research facilities, high-containment laboratories, and hospital sites.^1-13^ Building on the foundation of useful VHP data^2,14-16^ and subsequent approvals by the Food and Drug Administration since March 2020, the leadership at the state and institutional levels sought options for VHP decontamination locally. Our H_2_O_2_ decontamination process was founded on the FDA Emergency Use Authorization (EUA) granted to Battelle Memorial Institute^14^ and Duke University^3,16^ as well as statements and publications from other sites and agencies.^15,17-21^

At our institution, Campus Animal Resources (CAR) oversees the biomedical animal research operation and has used VHP in routine practice for many years. VHP exposures within the program have disinfected animal housing rooms between species and project cohorts and also decontaminated laboratory spaces with equipment like computers, incubators, texts, and lab notebooks without damaging effects.^22^ Building on our decontamination efforts to date, the VHP process for human medical equipment was assessed internally with the intent to return disinfected N95 respirators to their original owners at our hospital partner sites and assist safety groups (police and fire departments) with equipment disinfection across the state of Michigan. This study describes the intensive developmental process that was necessary to convert a multiroom animal housing facility into a regional VHP decontamination center in response to the impact of the coronavirus pandemic in the United States.

## Methods

### VHP System

Our VHP process uses the Halo Disinfection system, dispensed by the HaloFogger, and includes confirmation of VHP exposure within the area using chemical indicator H_2_O_2_ strip tests (Halosil, distributed by Quip Laboratories, Inc; Wilmington, DE). The HaloFogger disperses macrodroplets of 5% hydrogen peroxide solution, HaloMist fluid, in a uniform fashion for a timed period based on cubic footage of the room size, which then dictates the amount of PPE that can be decontaminated (Table 1). Biological efficacy of HaloMist has been confirmed by elimination of 6-log spore-forming (*Geobacillus stearothermophilus*; Spordex VH202 Biological Indicators [STERIS] and Apex, MesaLabs, Bozeman, MT) biological indicators (BI) incubated in media (Releasat, MesaLabs, Bozeman, MT) up to 7 days after completion of VHP cycle. HaloMist Disinfection System is EPA-registered (EPA No. 84526-6 Decision No. 510494; 6/10/16) and effective against *Clostridium difficile* spores as well as multiple bacterial species, viral agents, fungi, and prions.

**Table 1.** Individual Room Size, Vaporized Hydrogen Peroxide Cycle Parameters, and Mask Capacity Per Room within Facility.

Room No.	Floor Size (sq ft)	Room area (cubic ft)	Fog Time (min)	Dwell Time (min)	Exhaust (10–15 ach) Time (min)	Mask Capacity
10-13	202	1818	15	240	90	∼700
14-15	292	2628	18	240	90	1000
16-18	202	1818	15	240	90	700

ach = air changes per hour.

### Animal Facility

**Figure 1.**
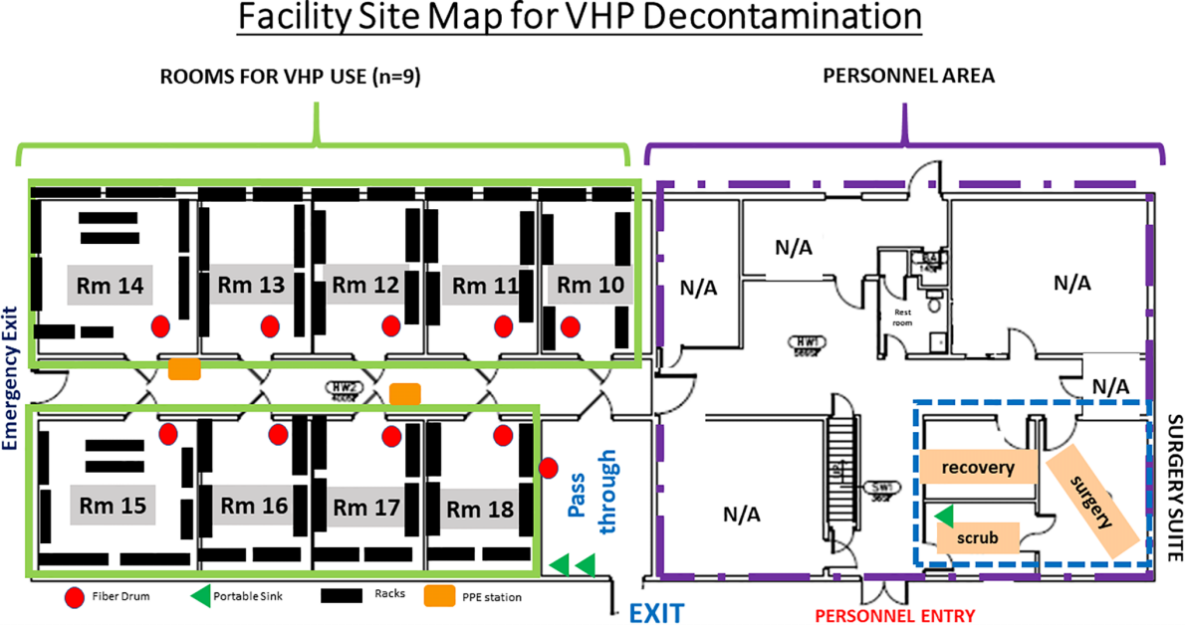
Building site map showing entry/exit doors, rooms, and surgical suite layout. After work within the facility, all staff are to exit through the pass-through, washing hands at portable sinks and discarding protective gear in this area before departure.

The selected animal housing facility (BLDG J) is a 10-room, 20 000-ft^2^ environmentally controlled stand-alone building. The facility accommodates housing and contains a 3-room animal surgery suite, restrooms, and break-room area; there is no public access, wet lab space, or connection to other facilities. The building HVAC is manually controlled and can be adjusted to meet VHP operation conditional requirements (temperature <70°F, humidity <50%). Animal housing rooms are temperature (68–72°F) and humidity (30–70%) controlled; within BLDG J in March/April, rooms are 68°F and ∼35% to 40% humidity. Due to the supply air needing to be shut off completely for the VHP cycles to function, temperatures are currently below that for which animal housing would be acceptable. The Animal Care Program and affiliated animal housing buildings are accredited by AAALAC. Facilities are inspected semiannually by the Institutional Animal Care and Use Committee, registered to the US Department of Agriculture (USDA), and are included in the Public Health Service Assurance for research use; importantly, this facility will not be used for animal housing during its conversion to a VHP disinfection center. Repurposing adjacent housing rooms (n = 9) allows for fogging of N95s in each space using 1 fogger per room; health teams enter through the converted surgical suite to don PPE (disposable gown, face protection, double gloves), and contaminated materials are delivered into the building through a separate entry that was formerly used as an emergency exit (Figure 1). Animal operations staff are to assess foggers and refill fluid reservoirs as needed, place chemical and biological indicators for each new cycle, stock PPE stations, and arrange for removal of full cardboard barrels for incineration, along with standard facility cleaning, as needed.

### Logistics of Building Operations

**Figure 2.**
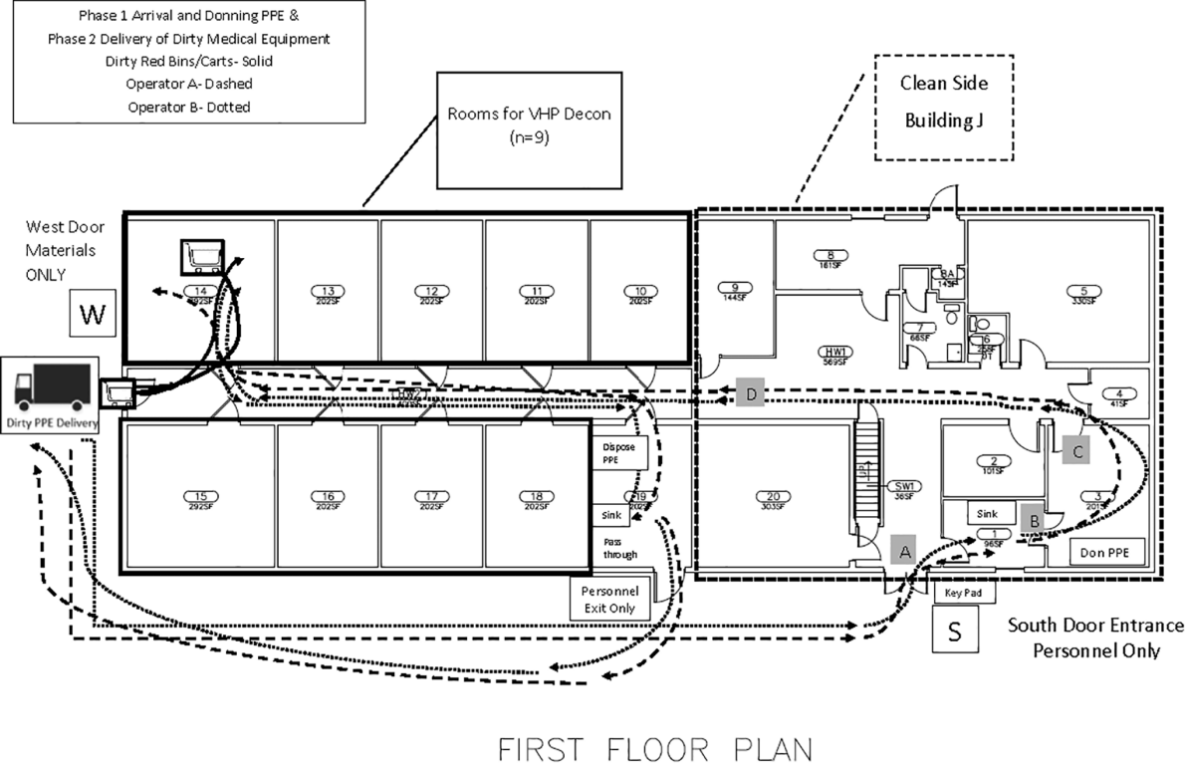
Process flow map for foot traffic of personnel separated from materials. Flow mapping was constructed onto the institutional site plan for building, utilizing a traditional emergency exit door (door W) to specifically set apart materials from personnel entry (door S). Health teams are expected to work in pairs, with 1 person entering the building, donning PPE, and then opening door W for their partner to pass over container(s) of used respirators. The partner then enters through door S, dons PPE, and meets the first team member at their assigned room to work together to unload N95s onto storage shelves.

Safety of personnel working within the facility was paramount; therefore, process flow maps were created using Microsoft Visio (Figure 2) to predict routes intended to limit crossover of labor, maximize social distancing, and separate the 2-person team from the soiled medical materials. The goal of the logistics effort was to ensure design of specific procedures so the facility remains (1) pathogen-free for all persons and (2) capable of safely processing medical items from regional hospitals for decontamination and return. Instructional signage was created to indicate personnel versus material entry, PPE donning and doffing, and stepwise information for positioning materials and operating the fogger; signage was laminated and posted throughout the facility on both the outside and inside of housing room doors. Centers for Disease Control and Prevention (CDC) PPE instructions for donning and doffing (https://www.cdc.gov/hai/pdfs/ppe/ppe-sequence.pdf) were posted throughout the rooms and delivered to health partners as part of their orientation materials.

### VHP Exposure to Unused N95 Respirators

Standard respirator styles used by institutional employees and by health partner sites were pilot-tested for repetitive exposure to VHP cycles to ensure that fit testing was not impacted. The following N95 respirators (1 of each) were removed from individual packaging, opened as if to be placed on the head, and laid dome up on storage racks. The respirators included: 3M 1860 (regular and small), 8110 (small), 8211 (regular), 1870+ (regular), 8511+ (regular), 9105vflex (regular and small), 9210+ (regular), and 9211+ (regular) and Moldex 1510 (extra small), 1511 (small), 1512 (medium), 2700 (medium/large), 2701 (small), 4200 airwave (medium/large), and 4201 airwave (small). After consecutive VHP exposures (n = 7) using the HaloFogger to deliver HaloMist as described earlier, all respirators were collected, individually bagged, and delivered to the environment, health, and safety (EHS) office for subsequent fit testing on trained EHS employees using the institution’s preferred quantitative method (TSI Portacount Respiratory Fit Tester, TSI). Filtration efficacy was inferred from studies by other sites that showed no issues with exposures up to 50 VHP cycles.^14^

### Biological Indicator Collection and Culture

VHP cycle parameters were tested repeatedly to ensure bacterial spore kill for all BIs. In brief, individual BIs are removed from packages by pulling open the edges of the pouch and laying the BI, without directly contacting the disk, into the bottom half of a plastic petri dish, with the domed side of the BI touching the dish. Petri dishes with BIs are placed at up to 10 distinct locations across storage racks, on high and low shelves, and at corners of the room most distant from the HaloFogger. Following completed VHP cycle (fog/dwell/exhaust) phases, petri dish bottoms are covered with corresponding tops, taped closed, and transported to an adjacent building for culture procedures. Sterile collection of BIs is critical to avoid cross-contamination; it is recommended to harvest BIs from petri dishes within a biosafety cabinet, using sterile forceps to place disks into media vials. Vials are to be sealed tightly and incubated (convection style incubator, Precision Scientific, set to 60°C) upright in test tube racks at 55°C to 60°C for up to 7 days. Each culture run should include a fresh BI that was not exposed to VHP, instead taken directly from its package and placed in media (control +). An unopened media vial (control –) is included with each culture.

### Training Instructions

Training documents and standard operating procedures were an essential component of the institutional FDA EUA application and captured each step of the process. In brief, health care/safety staff (in teams of two) inspect used N95 respirators for holes, tears, obvious damage, or significant visible soiling (including makeup, blood) and should discard these respirators to remove them from circulation at the originating site.

To ensure “chain of custody,” health care employees are instructed to label names on their used respirators with permanent marker and then place within individual paper bags before collection for decontamination. The batch of PPE destined for transfer to our institution is placed in biohazard containers (red bag style) that are closed and secured into plastic tote bins that are closed with a firm lid seal. CAR is contacted directly to schedule availability for VHP decontamination of N95s.

**Figure 3.**
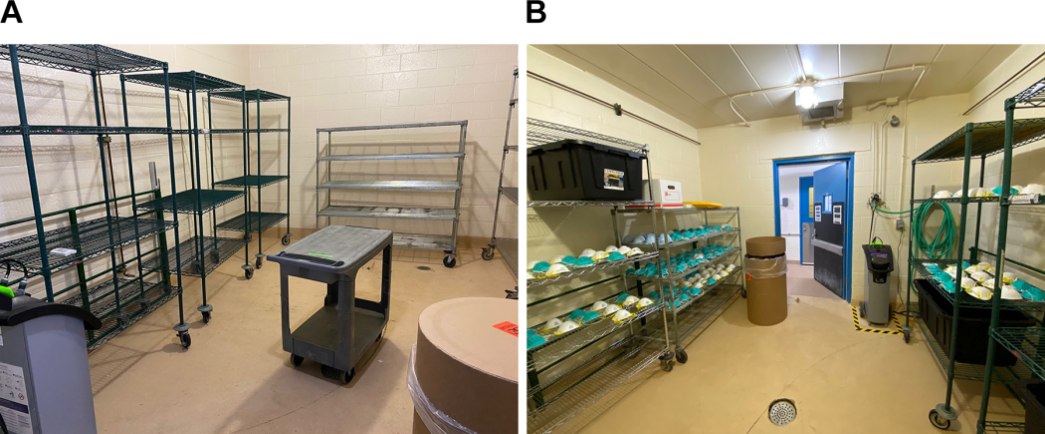
(A) Arrangement of empty housing room with racks, fogger, waste barrel, and hygrothermometer to record environmental parameters; (B) appearance of housing room with personal protective equipment after completion of vaporized hydrogen peroxide cycle at the time of biological indicator collection.

Health partner sites are asked to identify teams to serve as the dedicated N95 transporters/collectors, typically comprised of administrators and supply chain and surgery technicians with experience in donning/doffing PPE, sterile technique, and clean-to-dirty practices. Health teams are given a personal tour at the time of arrival prior to unloading any used materials within animal rooms. Health teams are instructed how to lay out N95s on storage racks in rooms, placing them with domed surface facing ceiling, and situate respirators to avoid overlap on storage rack shelving (Figure 3). Disposable items (respirators with makeup or stains, paper bags, packing materials, biohazard transport bags) are collected within the room into a cardboard fiber barrel lined with plastic for later incineration through institutional waste stream protocols. Prior to exit from rooms, health teams are to remove their outer layer of gloves, press the start button on the HaloFogger within the room, and exit to the hallway within 90 seconds before the initiation of fogging. Room doors are shut and frames sealed using 2-inch masking tape. To facilitate communication between health teams and CAR staff, the start time of the fogger and word *CYCLE* is to be written on a whiteboard placed on the outside of the room.

Staff exit the building through a pass-through room, distinct from where they and their materials entered, allowing for a different location to doff PPE per CDC guidelines and wash hands in portable sinks (borrowed from the institutional athletic department). Sanitizer in hands-free dispenser is provided in the pass-through area as an additional safety measure for use before departure.

### Postcycle Actions by Facility Staff

Once the appropriate fog/dwell phases are complete, animal operations staff enter the facility through the personnel pathway and verify that the chemical indicator placed on the interior of the room viewing window turned to the color black, confirming VHP exposure. (Note: In the rare circumstance when a viewing window indicator remains white, it will be assumed that the room has not been exposed; the VHP equipment will be assessed, and the fogger cycle will be run again.) Next, keeping the room door closed, the exhaust vent within the room is opened by using an exterior pull string that was positioned prior to VHP cycle start-up. Following the exhaust phase, facility staff remove masking tape from room door frame and stand at open doorway to check VHP levels (Drager X-am 5100 Single Gas H_2_O_2_ Monitor, handheld). To deem room safe for personnel entry, the H_2_O_2_ levels must be <1.0 ppm. If the room reading is higher than 1.0 ppm, the door should be closed, and more time should be allowed to evacuate vapor through the building exhaust system until the sensor reads below 1.0 ppm. BIs are collected, as described previously, without disturbing any of the arranged PPE. Next, CAR staff leave room, close door, and update whiteboard signage to read TEST with date and time to indicate that BIs are undergoing assessment. Facility staff exit building through the pass-through and deliver room BIs and positive control BI to laboratory (see “BI Collection and Culture Procedures”). Media tubes are assessed daily for up to 7 days, and health team personnel are contacted to arrange for PPE retrieval once bacterial kill is confirmed.

### Collection of Decontaminated Materials by Health Team

Similar to entry practices when bringing used materials to the building, health staff repeat these steps and maintain social distancing as the 2 team members don PPE and walk to their assigned room marked TEST. The health team enters the room and closes the door, then proceeds to gather PPE into clear plastic bagging (brought by the team) to clearly reveal name of original wearer. Each respirator is to have a hashmark placed with permanent marker along the respirator edge to verify 1 VHP exposure has occurred. Up to 20 VHP cycles are permitted for a single respirator, per recommendations from the FDA as part of the institutional EUA application. Items are placed within the transport containers that were left within the room to also be disinfected; the outer container is closed and sealed before leaving room. Health staff write “EMPTY” on the room door whiteboard, take the transport container with cleaned items to place outside the W door, and exit through the pass-through room. The transport container is retrieved, and the health team alerts the CAR team that the collection is complete. This final confirmatory communication allows the animal operations group to proceed to setup the housing rooms for a fresh VHP cycle in a timely manner.

### Functional Fit Tests

The hospital testing sites, as our partners in the design of this VHP decontamination project, return respirators to original wearers and conduct OSHA fit testing with their own employee health staff, including nurses, at their sites. Per OSHA announcements, VHP is a recognized method to decontaminate N95s for emergency use, in support of CDC directives on this method, with a number of caveats that are to be followed for wearing decontaminated respirators.^19,20,23^ Once an employee (regardless of whether from a health site or from the institution) is properly fit tested, the type and size of the N95 respirator is noted in a record that is kept in employee health/online training systems. Each respirator is to be inspected after the decontamination cycle, and respirators that are soiled, distorted, or perforated are discarded. Each health staff member will perform a self-seal test on their decontaminated respirator before use. If the respirator fails the self-seal test, it is discarded. Respirators for which the wearer has already been fit tested will be returned to the wearer and put through additional fit testing procedures (site-dependent). Hospital partners will use qualitative methods with either Bitrex (Moldex) or saccharine/bitter (3M) for fit testing.

## Results

Pilot tests of commonly used N95s exposed to consecutive cycles of VHP and then fit tested on employees were successful; no failed fit tests, no reported chemical odor, and no concerns about seals, elastic, and nose pieces were reported.

Pilot tests to verify VHP cycles were conducted without PPE (n = 8) and with used PPE (n = 5). Of those VHP trials conducted without PPE, the first 3 trials (data not shown) had more than 1 positive BI within 24 hours of culture; therefore, conditions continued to be optimized in consultation with Quip Laboratories. Of the next 5 trials (Table 2), only 1 had a positive BI when the disc was placed in closest proximity to the exhaust vent for the room. All other samples in those optimized trials without PPE had complete BI kill in media tubes incubated for up to 7 consecutive days. Once confidence in culture technique was solidified, contaminated PPE was brought to our facility by health care system partners (n = 3) and by safety responders involving police and fire departments (n = 2); all cultured BIs were successfully killed for these cycles, and materials were returned to their owners.

**Table 2.** Summary of Parameters of Trials to Establish VHP Cycles with and without PPE from Health and Safety Partners.

	4/13/20	4/15/20	4/15/20	4/17/20	4/17/20	4/20/20	4/21/20	4/23/20	4/30/20	4/30/20
Room	14	13	14	13	17	17	13	16	12	16
Room temp (°F)/humidity (%)	63/39	58/28	58/28	58/27	58/27	61/25	60/29	NR	67/50	65/51
Incubator temp (°C)	60	60	60	60	60	60	59	61	60	60
Biological indicator 6-log *Geobacillus* ^a^	S	S	S	A	S	S	S+A	S	S	S
BI locations^b^	1, 2, 3, 4, 5, 6, 7, 8	3, 4, 5, 6	3, 4, 5, 8	1, 2, 9, 10	1, 3, 6, 10	2, 3, 5, 10	2(S), 2(A), 3, 5(S), 5(A), 10	2, 3, 5, 10	1, 5, 7, 10	2, 3, 5, 10
Negative BI	7/8	4/4	4/4	4/4	4/4	4/4	5/5	4/4	4/4	4/4
Positive BI	Control + BI No. 2	Control	Control	Control	Control	Control	Control	Control	Control	Control
PPE present	No	No	No	No	No	Yes	Yes	Yes	Yes	Yes
Notes	BI No.2 positive: placed closest to exhaust vent			New shipment of BI tested		Health System A: ∼250 masks	Health System B: ∼200 masks; BI No. 10 lost in transit after cycle; new shipment of BI tested	Fire and police: ∼50 masks; room temp/humidity NR	Health System C: ∼200 masks	Fire and police: ∼100 masks

Abbreviations: A, apex; BI, biological indicators; NR, not recorded; PPE, personal protective equipment; S, spordex; VHP, vaporized hydrogen peroxide.

^a^ BI cultured in colorimetric media tubes (Steris; No. NA117, Lot PM-229); VHP cycle parameters for all trials: FOG (20 min) + DWELL (240 min) + EXHAUST (≥90 min).

^b^ BI locations: 1 (left side/lower shelf), 2 (left side/upper shelf), 3 (left rear/lower shelf), 4 (left rear/upper shelf), 5 (right rear/lower shelf), 6 (right rear/upper shelf), 7 (middle back/center shelf), 8 (middle front/center shelf), 9 (right side/lower shelf), 10 (right side/upper shelf).

Training documents created throughout the project included: instructions for health care facilities for VHP decontamination, instructions for health care personnel for VHP decontamination, health care provider fact sheet (FDA EUA format), instructions for facility operation of VHP decontamination equipment, and a standard operating procedure and data recording sheet on the placement and collection of BI in validating the VHP cycle. Dedicated data sheets recorded individual VHP cycle parameters and BI culture results for each pilot test; completed sheets were scanned and saved to a shared departmental server.

## Discussion and Conclusion

The ability to repurpose a secure vacant animal facility into a regional VHP decontamination center offered an unprecedented opportunity for veterinary, animal care, and medical professionals and administrators to cooperatively and creatively respond to the COVID-19 crisis. Every step in our protocol was tested by animal operations staff as well as logistics experts, medical professionals, and administrators at Health Systems A and B. Health System A contains 5 hospitals serving seven counties, and Health System B has 5 acute care hospitals and 2 psychiatric hospitals, overall serving >15 counties in the state. Between these 2 major hospital systems, more than 18 000 clinicians and support staff are providing care and services to patients suspect for and confirmed with COVID-19. Although both hospital systems have supply chain leaders and purchasing staff working tirelessly to obtain requisite PPE, on any given day, the supplies for isolation gowns, face shields, and N95s are dire. The estimated “burn rate” considering the single use of respirators ranges from 2300 to 3200 per day. They estimate the burn rate for isolation gowns ranges from 13 000 to 18 000 per day. Our regional decontamination site is intended to serve as a stopgap measure to offset the discard of single-use PPE until such time that suppliers are secured to restore ready access to PPE at these health centers.

Important to the COVID-19 response, virucidal activity of VHP is paramount, and the ability to kill *Geobacillus* spores serves as a proxy to ensure that environmentally hardy organisms are eliminated by the cycle time described for the Halofogger with HaloMist.^24^ We did not have access to confirmed COVID-19 samples to test eradication of coronavirus by the hydrogen peroxide fogging system; however, the EPA data for this chemical confirm virucidal activity. One of the major advantages of hydrogen peroxide is its safety profile; it readily breaks down into water and oxygen with no toxic by-products or residues. Our cycle design includes a dwell phase that is double the length of the manufacturer recommended time, and after a 90-minute exhaust phase (during which the room air changes per hour are minimally 10–15 per hour), there has not been detection of H_2_O_2_ levels above 1.00 ppm remaining in the room at the time of BI collection. Importantly, safety of personnel working within the facility is paramount, and process flow mapping has been executed to ensure limited crossover of labor, social distancing, and appropriate donning and doffing of protective gear for these needs. The shared partnership between health care and facility staff eliminates concerns about labor fatigue for any group working on this process.

Because of the repurposing of an animal facility for this request and given the number of higher education/biomedical universities that have similar animal facilities at their locations, this project is readily adaptable for similar innovation at multiple institutions throughout the United States, a great many of which already have access to and ownership of portable H_2_O_2_ fogging devices. Institutions with AAALAC-accredited animal research areas, all of which comply with regulated environmental controls, will be able to use our protocols, specific VHP cycle times, and BI exposure and collection instructions to deliver VHP decontamination of used medical materials for their regions as well.

Our institution is actively seeking FDA EUA approval because this regional site has the ability to influence multiple areas throughout the state, in particular to sterilize N95 respirators for reuse by the original wearer and disinfect other medical items (face shields, eyewear, and isolation gowns) that may potentially become challenging to procure in the weeks and months ahead. We believe that the use of this dedicated facility for H_2_O_2_ decontamination will support multiple larger clinical and smaller safety units and their essential employees for all first-responders to rely confidently on the security and efficacy of their PPE while they give care to patients and the public.
